# Clinical and Microbiological Characterization of Carbapenem-Resistant *Enterobacteriales*: A Prospective Cohort Study

**DOI:** 10.3389/fphar.2021.716324

**Published:** 2021-10-08

**Authors:** Qiuxia Lin, Menglu Wu, Hanbing Yu, Xiaojiong Jia, Hua Zou, Deyu Ma, Siqiang Niu, Shifeng Huang

**Affiliations:** ^1^ Department of Clinical Laboratory Medicine, The First Affiliated Hospital of Chongqing Medical University, Chongqing, China; ^2^ Department of Clinical Laboratory, Qingdao Women and Children’s Hospital, Qingdao, China; ^3^ Department of Clinical Laboratory Medicine, Chongqing Health Center for Women and Children, Chongqing, China

**Keywords:** mortality, carbapenemase-producing *Klebsiella pneumoniae*, carbapenemase-producing *Enterobacteriales*, multilocus sequence typing, molecular epidemiology

## Abstract

**Aim:** We aim to depict the clinicoepidemiological and molecular information of carbapenem-resistant *Enterobacteriales* (CRE) in Chongqing, China.

**Methods:** We performed a prospective, observational cohort study, recruiting inpatients diagnosed with CRE infections from June 1, 2018, to December 31, 2019. We carried out strain identification and molecular characterization of CRE. eBURST analysis was conducted to assess the relationships among the different isolates on the basis of their sequence types (STs) and associated epidemiological data using PHYLOViZ. Clinical parameters were compared between the carbapenemase-producing *Enterobacteriales* (CPE) and non-CPE group.

**Findings:** 128 unique CRE isolates from 128 patients were collected during the study period: 69 (53.9%) CPE and 59 (46.1%) non-CPE. The majority of CPE isolates were *bla*_KPC-2_ (56.5%), followed by *bla*_NDM_ (39.1%) and *bla*_IMP_ (5.8%). *Klebsiella pneumoniae* carbapenemase (KPC)–producing clonal group 11 *Klebsiella pneumoniae (K. pneumoniae)* was the most common CPE. Antibiotic resistance was more frequent in the CPE group than in the non-CPE group. Independent predictors for CPE infection were ICU admission and hepatobiliary system diseases. Although, there was no significant difference in desirability of outcome ranking (DOOR) outcomes between the two groups. At 30 days after index culture, 35 (27.3% ) of these patients had died.

**Conclusion:** CRE infections were related to high mortality and poor outcomes, regardless of CRE subgroups. CPE were associated with prolonged ICU stays and had different clinical and microbiological characteristics than non-CPE. The identification of CPE/non-CPE and CRE resistance mechanisms is essential for better guidance of the clinical administration of patients with CRE infections.

## Introduction

Antimicrobial resistance (AMR) poses a serious threat to global health, economics, and medical practice, and the resistance to last-line antibiotics (i.e., carbapenems) is of most concern (World Bank Group (2017); WHO Antimicrobial resistance, 2014). Moreover, CRE belong to the top three multidrug-resistant (MDR) bacteria on the priority list of the WHO (Tacconelli et al., 2018). According to the definition of the United States Centers for Disease Control and Prevention (CDC), *Enterobacteriales* with resistance to a carbapenem are defined as CRE (CRE, 2015). Generally, CRE are divided into carbapenemase-producing *Enterobacteriales* (CPE) and non-CPE groups. In virtue of the quick spread of CPE in health-care settings and high mortality rates, CPE infections have attracted high clinical and public health attention (Haverkate et al., 2015).

Carbapenem resistance within *Enterobacteriales* derives from two main mechanisms: 1) acquisition of carbapenemase genes that encode enzymes capable of hydrolyzing carbapenems or 2) a decrease in the uptake of antibiotics by a qualitative or/and quantitative deficiency of porin expression in association with overexpression of β-lactamases that possess very weak affinity for carbapenems (Nordmann et al., 2012).Globally, the most important carbapenemases of carbapenem resistance within *Enterobacteriales* are classified into three categories: 1) class A serine enzymes, such as KPC-type enzymes; 2) class B metal enzymes, such as NDM, VIM, and IMP metallo-β-lactamases, and 3) class D serine enzymes, such as OXA-48–type enzymes (Nordmann et al., 2012). In some recent observational studies from Italy, Greece, Spain, and the United States, high mortality rates (40–60%) in patients with bacteremia caused by CPE were observed (Zarkotou et al., 2011; Qureshi et al., 2012; Tumbarello et al., 2012; Navarro-San Francisco et al., 2013; Daikos et al., 2014). In another observational study in 2013, the predominant species of CPE in the United States was *K. pneumoniae* (Satlin et al., 2017). Moreover, KPC is the most common carbapenemase in the United States (CDC, 2021). NDM-1 was widespread in India, Pakistan, and Bangladesh (Kumarasamy et al., 2010). OXA-48–like carbapenemases remain extremely rare in the United States; on the contrary, they are relatively commonly found in Europe, especially in Mediterranean countries (Poirel et al., 2012; Lyman et al., 2015). Carbapenemases have a worldwide distribution, but substantial variability in the distribution of carbapenemases exists on the continental, national, and regional levels. Most clinicoepidemiological information and its association with molecular characteristics have been reported for Western countries, including those in North America and/or Europe, and have focused on molecular types such as KPC or NDM. Awareness of the prevalence and incidence of the specific carbapenemases of CRE is essential for the prevention of their spread and selection of appropriate treatment options. To better assist the clinicians in administration of CRE infections, more detailed clinicoepidemiological information and its association with molecular characteristics are highly needed. In this prospective study, our research question was whether carbapenemase production in CRE is associated with adverse clinical outcomes, among CRE in China.

## Materials and Methods

### Study Settings and Participants

We performed a single-centre, prospective, observational cohort study at a tertiary-care, teaching hospital in Chongqing, China. Patients were eligible for inclusion if CRE was isolated in a clinical culture from any anatomical site during hospitalization. Institutional review board approval was obtained, and only the first qualifying culture episode during the first admission for each unique patient enrolled during the study period (June 1, 2018, to December 31, 2019) with CRE infection was included. There was no age exclusion. We excluded patients who had been involved in this study before and who refused treatment at the onset of infection. The study size was derived by inclusion of all eligible patients within the study period. Infections were defined by previously described standard criteria (Van Duin et al., 2014). A patient with a culture positive for CRE was deemed to have an infection if CRE was isolated from blood or any other sterile source. For patients with positive respiratory cultures, the criteria outlined by the American Thoracic Society and the Infectious Diseases Society of America were used (Pneumonia, 2005; Mandell et al., 2007). For patients with positive cultures from urine or surgical wounds, the CDC National Healthcare Safety Network criteria were applied (Horan et al., 2008). Patients with cultures from nonsurgical wounds were considered infected only if the treating physician documented infection in the medical record and evidence of systemic inflammation on the day the positive culture was documented; this was defined as an abnormal systemic white cell count (either > 10 × 10^3^ or < 4 × 10^3^ cells/μl) and/or an abnormal body temperature (either > 99.5°F or < 96°F). All of the other culture episodes were designated colonizations. According to CDC guidelines for clinical microbiology laboratories, CRE are defined as *Enterobacteriales* with resistance to any carbapenem (meropenem, imipenem, and/or ertapenem) and/or phenotypically carbapenemase producers (CRE, 2015). Follow-up time: from admission to discharge or death in hospital. Those discharged alive were followed up until 90 days after the first positive culture.

### Clinical Data Collection

Clinical data, including demographics (age and sex), referral source (admission from another hospital, nursing home, long-term care facility, or home), isolation source of bacteria (e.g., blood and bronchial lavage fluid), severity of comorbid conditions (recorded by the Charlson comorbidity index (CCI) score (Charlson et al., 1987)), the Pitt bacteraemia score (Paterson et al., 2004) on the day of the first culture, exposure to antibiotic within 14 days before the first positive culture collection date of CRE, history of trauma and surgical procedures during 6 months before the CRE culture collection date, antibiotic usage of empirical therapy, antibiotic usage of definitive therapy, and outcomes, were collected from the patients’ electronic medical records system. Empirical therapy was defined as antibiotic usage before the antibiotic susceptibility report. Definitive therapy was antibiotics given after the antibiotic susceptibility report.

### Microbiology

Standard identification and susceptibility testing of CRE were carried out and interpreted, according to the Clinical and Laboratory Standards Institute (CLSI) criteria of microbiology laboratories (Clinical Laboratory Standards Institute, 2018). Bacterial identification was conducted by using the VITEK MS or VITEK2 compact (BioMerieux, Hazelwood, MO, United States) automated system, and antimicrobial susceptibilities were determined *in vitro* using a VITEK 2 Compact AST-GN13 card (bioMérieux), which was used to test the antibiotic susceptibilities of all isolates to cefazolin (CFZ), ceftriaxone (CRO), ceftazidime (CAZ), cefepime (FEP), cefoxitin (FOX), aztreonam (ATM), ampicillin/sulbactam (SAM), piperacillin/tazobactam (TZP), ciprofloxacin (CIP), levofloxacin (LEV), minocycline (MH), and cefoperazone/sulbactam (CFPS). Four antibiotics, including imipenem (IMP), meropenem (MEM), ertapenem (ETP), and tigecycline (TGC), were tested by the broth microdilution method following the criteria of the CLSI (Clinical Laboratory Standards Institute, 2018). The minimal inhibitory concentrations (MICs) breakpoint for tigecycline was defined according to the European Committee on Antimicrobial Susceptibility Testing (EUCAST, 2021), while the others were interpreted according to CLSI protocols. Using the previously described primers (Tian et al., 2018), polymerase chain reaction (PCR) amplification of genomic DNA extracted from CRE isolates and subsequent PCR amplification product sequencing were performed to identify potential carbapenemase genes (i.e., *bla*_KPC_, *bla*_NDM_, *bla*_IMP_, *bla*_VIM_, and *bla*_OXA-48_) (Wang et al., 2018), extended spectrum-β-lactamases (ESBLs) genes (i.e., *bla*_CTM_, *bla*_SHV_, and *bla*_TEM_), and AmpC genes (i.e., *bla*_ACC_, *bla*_FOX_, *bla*_*MOX*_, *bla*_*DHA*_, *bla*_*CIT*_, and *bla*_*EBC*_). Multilocus sequence typing (MLST) was conducted following the published protocol for *K. pneumoniae* (http://bigsdb.pasteur.fr/klebsiella/klebsiella.html). eBURST analysis was conducted to assess relationships between the different isolates on the basis of their sequence types (STs) and associated epidemiological data using PHYLOViZ.

### Outcomes

After 30 days of the first positive culture, we evaluated the outcome. The primary outcome of this study was DOOR (Evans et al., 2015) analysis evaluating three harmful events (discharge failure, adverse events, and absence of clinical response, Supplementary Appendix). Death and being alive without harmful events were, respectively, defined as the worst and the best outcome. Between the two extremes, the three categories were alive with one, two, or three harmful events. There were five total categories of outcomes. The secondary outcomes of our study were in-hospital mortality, 30-day mortality, 90-day mortality, and 90-day readmission rates.

### Statistical Analysis

Characteristics of patients with CPE infection were compared to those with non-CPE infection using Fisher’s exact tests or chi-square for categorical variables and Mann–Whitney U-tests for continuous variables as appropriate. Univariate analyses were performed for each variable. Variables with *p* value of ≤0.1 in the univariate analyses were included into the multivariate logistic regression model. All *p* values were two-sided, and *p* values ≤0.05 were considered statistically significant. All statistical analyses were conducted by IBM SPSS software version 25.0 (SPSS Inc., IL, United States).

## Results

### Summary of the Study Cohort

During the study period, a total of 128 isolates were collected from 128 patients (Supplementary Table S1). Carbapenemase genes were shown in 69 (53.9%) strains, referred to as CPE. On the other hand, 59 (46.1%) CRE resistant to at least one carbapenem, without any carbapenemase gene, were defined as non-CPE.

### Bacterial Species and Isolation Sites

In Supplementary Table S1, bacterial species of CRE are summarized. CRE isolates included 69 (53.9%) *K. pneumoniae*, 24 (18.8%) *Enterobacter spp*., 20 (15.6%) *Escherichia coli (E. coli)*, 7 (5.5%) *Klebsiella aerogenes (K. aerogenes)*, 5 (3.9%) *Citrobacter freundii (C. freundii)*, 2 (1.6%) *Klebsiella oxytoca (K. oxytoca),* and 1 (0.8%) *Enterobacter kobei (E. kobei)*. For CPE isolates, the predominant genus and species were *K. pneumoniae* (60.7%), *Enterobacter spp*. (20.3%), and *E. coli* (11.6%); there were two (2.9%) *C. freundii* and one (1.4%) isolate of *K. aerogenes, K. oxytoca,* and *E. kobei*. For non-CPE, the genus and species distribution was largely similar but slightly reverse: *K. pneumoniae* (45.8%), *E. coli* (20.3%), *Enterobacter spp*. (16.9%), *K. aerogenes* (10.2%), *C. freundii* (5.1%), and *K. oxytoca* (1.7%).

In Supplementary Table S2, isolation sites of CRE are summarized. CPE were most commonly isolated from sputum (*n* = 27, 39.1%), followed by urine (*n* = 16, 23.2%), blood (*n* = 11, 15.9%), drainage (*n* = 6, 8.7%), wound secretion (*n* = 4, 5.8%), bile (*n* = 2, 2.9%), and others (*n* = 1, 1.4%). Non-CPE were most commonly isolated from urine (*n* = 18, 30.5%), followed by sputum (*n* = 13, 22.0%), drainage (*n* = 8, 13.6%), wound secretion (*n* = 7, 11.9%), blood (*n* = 5, 8.5%), and catheter (*n* = 4, 6.8%), bile (*n* = 3, 5.1%), intra-abdominal samples (*n* = 2, 2.9%), and others (*n* = 1, 1.7%).

### Antimicrobial Susceptibility Profiles

Among non-β-lactam antibiotics, levofloxacin had significantly higher susceptibility rates in non-CPE [18 (30.5%)] than in CPE [8 (11.6%)], *p* = 0.008 in Supplementary Table S3. Similarly, among β-lactams, imipenem [non-CPE/CPE: 27(45.8%)/2(2.9%)], meropenem [30(50.9%)/3(4.3%)], piperacillin/tazobactam [10(16.9%)/0], cefepime [7(11.9%)/0], and cefoperazone/sulbactam [7(11.9%)/0] had significantly higher susceptibility rates in non-CPE than in CPE. Overall, CPE had higher resistant rates in those agents.

### Prevalence of Carbapenemase, ESBLs, and AmpC Among CRE

Detected carbapenemase genes included *bla*_KPC-2_ (39 [56.5%] of 69), *bla*_NDM-1_ (19 [27.5%]), *bla*_NDM-5_ (8 [11.6%]), and *bla*_IMP-4_(4 [5.8%]); ESBL genes shown in CPE included *bla*_CTX-M_ (13 [18.8%] of 69) and *bla*_SHV_ (3 [4.3%]). Non-CPE showed similar *bla*_CTX-M_ (14 [23.7%] of 59) and *bla*_SHV_ (3 [5.1%] of 59) distribution characteristics. There are no differences in AmpC expression between CPE (5 [7.2%] of 69) and non-CPE (3 [5.1%] of 59). Notably, a *K. aerogenes* isolate simultaneously coproduced *bla*_NDM-1_, *bla*_KPC-2_, and *bla*_CTX-M_ (Table 1). 42 (60.9%) of the 69 carbapenem-resistant *K. pneumoniae* isolates were CPE, compared with 14 (58.3%) of 24 for *Enterobacter spp*. and 8 (40.0%) of 20 for *E. coli*. Among 69 CR-KP isolates, *bla*_KPC-2_ was the most commonly identified carbapenemase-encoding gene; it was present in 37 CR-KP isolates. *Bla*_IMP-4_ was shown in 3 CR-KP isolates and *bla*_NDM-5_ in 2 CR-KP isolates. Among 14 carbapenemase-producing *Enterobacter spp*. isolates, all carbapenemase-encoding genes were *bla*_NDM-1_. However, various metallo-β-lactamase genes were identified in 8 carbapenemase-producing *E. coli*, including *bla*_NDM-1_ (1 of 8), *bla*_NDM-5_ (6 of 8), and *bla*_IMP-4_ (1 of 8) (Supplementary Table S4).

**TABLE 1:** Prevalence of carbapenemase, ESBL, and AmpC among CRE.

	CPE (*n* = 69)	Non-CPE (*n* = 59)	Total (*n* = 128)	*p* value
Carbapenemase genes			
*bla*_kpc-2_	39 (56.5%)	-	39 (30.5%)	
*bla*_NDM-1_	19 (27.5%)	-	19 (14.8%)	
*bla*_NDM-5_	8 (11.6%)	-	8 (6.3%)	
*bla*_IMP-4_	4 (5.8%)	-	4 (3.1%)	
Extended spectrum β-lactamase genes			
*bla*_CTX-M_	13 (18.8%)	14 (23.7%)	27 (21.1%)	0.499
*bla*_SHV_	3 (4.3%)	3 (5.1%)	6 (4.7%)	>0.999
AmpC	5 (7.2%)	3 (5.1%)	8 (6.3%)	0.725

*Data are expressed as n (%).*

A *K. aerogenes* isolate was simultaneously coproduced *bla*_NDM-1_, *bla*_KPC-2_, and *bla*_CTX-M_

### MLST of *K. pneumoniae* Isolates

**FIGURE 1:**
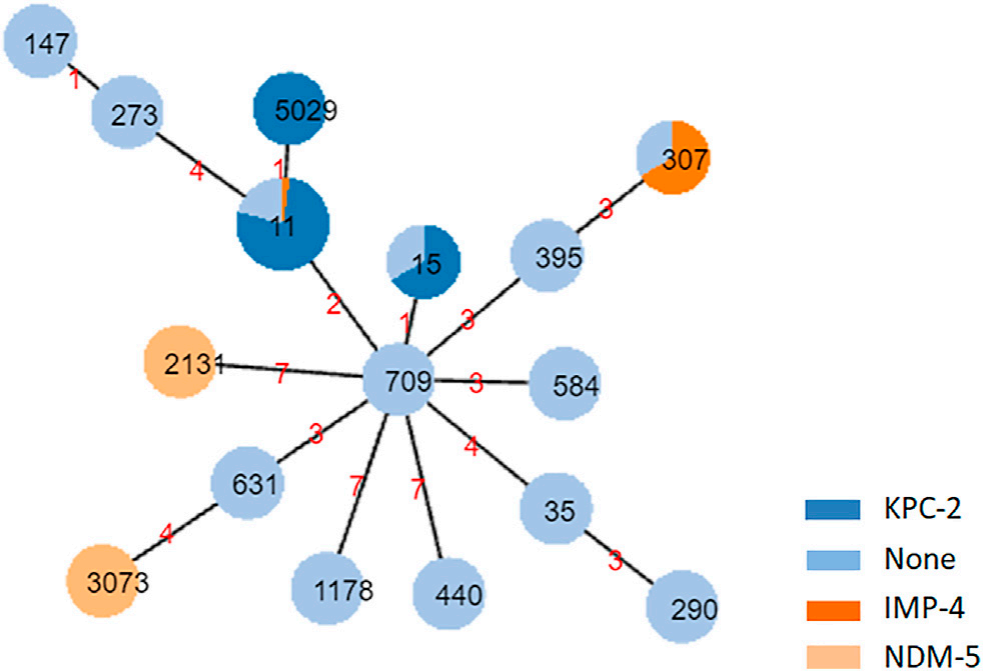
eBURST diagrams of *Klebsiella pneumoniae* isolates showing the relationship among isolates on the basis of their multilocus sequence typing and carbapenemase. Each node within the tree represents a single ST. The size of the nodes is proportional to the number of isolates represented by the said node. Within each node, the area of dark blue, dark orange, light orange, and light blue represents the number of KPC-2, IMP-4, NDM-5 and no carbapenemase. Nodes are labeled with corresponding ST. ST, sequence type. Links are labeled with absolute distance.

In total, 69 *K. pneumoniae* isolates were subjected to MLST. We have identified 16 *K*. *pneumoniae* STs. The most common ST of *K*. *pneumoniae* was ST11 (43/69, 62.3%). The most frequent carbapenemase type and ST in *K. pneumoniae* was ST11-KPC-2 *K. pneumoniae*(33/69, 47.8%), followed by ST15-KPC-2 *K. pneumoniae* and ST307-IMP-4 *K. pneumoniae* (two of each type). eBURST diagrams based on the STs obtained for *K. pneumoniae* are shown by carbapenemase(Figure 1). 48 isolates belonged to clonal complex 11, including ST11, ST15, ST709, and ST5029. Three isolates belonged to clonal complex 307, including ST307. Three isolates belonged to clonal complex 147, including ST147 and ST273. Nine STs were uniquely represented by one isolate. In addition, the ST of remaining 6 *K. pneumoniae* isolates was not identified (Table 2).

**TABLE 2:** The MLST analysis of 69 CR-KP.

	CPE			Non-CPE
	KPC-2	NDM-5	IMP-4	
	ST11 (33)	ST2131(1)	ST11(1)	ST11 (9)
**ST**	ST15 (2)	ST3073(1)	ST307(2)	ST147(2)
	ST5029 (1)			Other types^a^ (11)
	Unknown ST (1)			Unknown ST (5)

a Other types: ST709, ST631, ST584, ST440, ST395, ST35, ST307, ST290, ST273, ST15, and ST1178. ST, sequence type.

### Comparison of Clinical Characteristics and Outcomes of CPE and Non-CPE

Table 3 showed the results of the univariate analysis of the comparison between patients with CPE infections and those with non-CPE infections. For the univariate analyses, there were no significant differences in demographic information and most preexisting medical conditions between the two groups. 50 (39.1%) of the 128 patients showed a history of intensive care unit (ICU) admission. As compared with patients with non-CPE infections (16 [27.1%] of 59), patients with CPE infections (34 [49.3%] of 69) were more likely to be with the history of ICU admission. Moreover, patients with CPE infections tended to have longer ICU stay and hepatobiliary system disease than patients with non-CPE infections. Among the patients with CRE infections, patients with CPE infections represented a greater likelihood of having respiratory tract infections (27 [39.1%] of 69; *p* = 0.038) and lower likelihood of having catheter-related infections (0 of 69; *p* = 0.028) rather than patients with non-CPE infections (respiratory 13 [22.0%] of 59; catheter-related 4 [6.8%] of 59; for overall distribution, *p* = 0.042). Multivariate analysis for matched data showed that admission to ICU (OR, 5.55; 95% CI, 2.25–13.74; *p* < 0.001) and hepatobiliary system disease (OR, 2.91; 95% CI, 1.27–6.66; *p* = 0.011) were independent predictors for CPE isolation of patients with infection (Table 4).

**TABLE 3:** Baseline characteristics of patients with CRE infection.

Variables	CPE (*n* = 69)	Non-CPE (*n* = 59)	*p* value
Age, years	64(53–78)	62(54–74.5)	0.833
Sex			0.873
Male	50(72.5%)	42(71.2%)	
Female	19(27.5%)	17(28.8%)	
aCCI	4(2–6)	4(2.5–7)	0.466
Pitt bacteraemia score	2(0–2)	1(0–2)	0.357
Time to positive culture, days	9(2.5–21)	9(2–21)	0.770
Community onset	21(30.4%)	19(32.2%)	0.830
Admitted from home	36(52.2%)	38(64.4%)	
Hospital transfer	33(47.8%)	21(35.6%)	0.162
ICU admission	34(49.3%)	16(27.1%)	**0.01**
The length of ICU stay, days	22(8.5–31.5)	10.5(5.25–26)	**0.045**
Antimicrobial exposure	60(87.0%)	52(88.1%)	0.841
Surgery	30(43.5%)	32(54.2%)	0.225
Transplant history	2(2.9%)	3(5.1%)	0.525
Multiple organ dysfunction	19(27.5%)	9(15.3%)	0.198
History of blood transfusion	28(40.6%)	18(30.5%)	0.296
Chemotherapy	5(7.2%)	6(10.2%)	0.556
Radiotherapy	0(0.0%)	1(1.7%)	0.272
Tumor	24(34.8%)	23(39.0%)	0.623
Infection			
Blood infection	11(15.9%)	5(8.5%)	0.203
Urine infection	16(23.2%)	18(30.5%)	0.350
Respiratory infection	27(39.1%)	13(22.0%)	**0.038**
Wound infection	4(5.8%)	7(11.9%)	0.222
Intra-abdominal infection	0(0.0%)	2(3.4%)	0.187
Catheter-related infection	0(0.0%)	4(6.8%)	**0.028**
Other infections	9(13.0%)	12(20.3%)	0.267
Septic shock	22(31.9%)	11(18.6%)	0.088
Intrusive procedure			
Drainage tube	38(55.1%)	44(74.6%)	**0.022**
Urinary catheter	44(63.8%)	48(81.4%)	**0.027**
Venous catheterization	41(59.4%)	36(61.0%)	0.854
Arterial catheterization	28(40.6%)	21(35.6%)	0.563
Mechanical Ventilation	33(47.8%)	25(42.4%)	0.537
Tracheal intubation	28(40.6%)	21(35.6%)	0.563
Tracheotomy	18(26.1%)	8(13.6%)	0.079
Bronchoscope	12(17.4%)	11(18.6%)	0.54
Underlying disease			
Respiratory system diseases	45(65.2%)	29(49.2%)	0.067
Urinary system diseases	22(31.9%)	22(37.3%)	0.521
Cardiovascular diseases	40(58.0%)	31(52.5%)	0.538
Cerebrovascular disease	19(27.5%)	18(30.5%)	0.712
Immune system diseases	5(7.2%)	2 (3.4%)	0.339
Blood system diseases	26(37.7%)	16(27.1%)	0.205
Central nervous system disease	11(15.9%)	9(15.3%)	0.915
Endocrine system diseases	9(13.0%)	10(16.9%)	0.536
Hepatobiliary system disease	42(60.9%)	25(42.4%)	**0.037**
Gastrointestinal disease	24(34.8%)	25(42.4%)	0.379
Urinary system disease	33(47.8%)	22(37.3%)	0.230
Peripheral vascular disease	14(20.3%)	11(18.6%)	0.815

Data are expressed as n (%) of patients for categorical variables and median (IQR) for continuous variables as appropriate. p values of ≤0.05 were presented by bold values.

**TABLE 4:** Logistic regression analysis of predictors factors for CPE.

	OR(95% CI)	*p* value
ICU admission	5.55(2.25–13.74)	**<0.001**
Drainage tube	0.28(0.10–0.75)	**0.011**
Hepatobiliary system disease	2.91(1.27–6.66)	**0.011**

OR, odds ratio; CI, confidence interval; ICU, intensive care unit. p values of ≤0.05 were presented by bold values.

Antibiotic treatment history is displayed in Supplementary Table S5. 61 (47.5%) of 128 patients received empirical therapy with a carbapenem, while 57 (44.5%) of the 128 patients received definitive therapy with a carbapenem. In empirical therapy, 18 patients received monotherapy with a carbapenem. Also, in definitive therapy, 8 patients received monotherapy with a carbapenem. There were no significant differences between two groups in most agents of empiric and directed antibiotic regimens. Patients with CPE infection received more cefoperazone-sulbactam (29.0% vs. 11.9%, *p* = 0.018), less aminoglycoside (29.0% vs. 45.8%, *p* = 0.050), and more tigecycline (36.2% vs. 18.6%, *p* = 0.027) than patients with non-CPE infection in definitive therapy.

According to DOOR outcomes shown in Table 5, 37 (28.9%) patients with CRE infections were alive without events, 31 (24.2%) with one event, 19 (14.8%) with two events, 6 (4.7%) with three events, and 35 (27.3%) were dead. Between patients with CPE infections and with non-CPE infections groups, there was no significant difference of DOOR outcomes. In patients with CRE infections, 30-day mortality and 90-day mortality were, respectively, 27.3% (35 of 128) and 32.8% (42 of 128). Between the CPE and non-CPE groups, 30-day mortality and 90-day mortality rates were similar 25 (19.5%) were readmitted within 90 days. There were no significant differences between the two groups.

**TABLE 5:** Outcomes in patients with CRE infections.

	CPE (*n* = 69)	Non-CPE (*n* = 59)	Total (*n* = 128)	*p* value
DOOR at 30 days				0.180
Alive without events	17(24.6%)	20(33.9%)	37(28.9%)	
Alive with one event	16(23.2%)	15(25.4%)	31(24.2%)	
Alive with two events	11(15.9%)	8(13.6%)	19(14.8%)	
Alive with three events	4(5.8%)	2(3.4%)	6(4.7%)	
Death	21(30.4%)	14(23.7%)	35(27.3%)	
DOOR components at 30 days				
Not discharged	31(44.9%)	24(40.7%)	55(43.0%)	0.628
Lack of clinical response	43(62.3%)	33(55.9%)	76(59.4%)	0.463
Renal failure	13(18.8%)	9(15.3%)	22(17.2%)	0.592
Total LOS, days	30(16–52)	26(15–50)	27(15–50.75)	0.261
LOS after isolation^a^, days	20(11–32.5)	16(8–26)	14.5(7–26)	0.220
30-day mortality	21(30.4%)	14(23.7%)	35(27.3%)	0.396
90-day mortality	26(37.7%)	16(27.1%)	42(32.8%)	0.205
90-day readmissions	16(23.2%)	9(15.3%)	25(19.5%)	0.259
Clinical response	26(37.7%)	25(42.4%)	51(39.8%)	0.589
Disposition				0.444
Death	26(37.7%)	16(27.1%)	42(32.8%)	
Home	42(60.9%)	42(71.2%)	84(65.6%)	
Long-term care	1(1.4%)	1(1.7%)	2(1.6%)	

Data are expressed as n (%) of patients for categorical variables and median (IQR) for continuous variables as appropriate.

DOOR, desirability of outcome ranking; LOS, length of hospital stay.

a Excluding cases who died in hospital.

## Discussion

We aim to depict the clinicoepidemiological and molecular information of CRE in China. In this study, CRE were divided into two clinically and molecularly distinct groups (CPE and non-CPE groups). While CPE are *Enterobacteriales* that are resistant to carbapenem and express carbapenemase, non-CPE are CRE without any carbapenemase genes. In general, CPE are considered to be of the most epidemiological significance due to their ability to spread rapidly throughout health-care settings and their association with adverse outcomes. Nevertheless, in our cohort, 46.1% of the isolates did not carry carbapenemase genes. Resources dedicated to stopping the spread of CPE isolates might, therefore, be assigned to non-CPE of lesser public health concern. Therefore, correct identification of CPE is essential for the management of CRE infections. This study provided important information including the molecular microbiological characteristics of CPE and non-CPE groups, as well as clinical epidemiology and the differences between the two groups and predictors for CPE isolation and outcomes.

Among 128 CRE isolates, CR-KP was the most dominant species (53.9%), followed by *Enterobacter spp*. (18.8%) and *E. coli* (15.6%). *bla*_KPC-2_ was the most commonly identified carbapenemase-encoding gene. Among 69 CR-KP, most isolates belonged to clonal complex 11, including ST11, ST15, ST709, and ST5029. ST11 was the most common ST type of CR-KP in our study, consistent with a multicenter study in China (Wang et al., 2018). In another study, the dominant ST of CR-KP was, and still is, ST258, which has become prevalent in many places around the world since it was first discovered in the United States (Kitchel et al., 2009; Munoz-Price et al., 2013). The most frequent carbapenemase type and ST in CR-KP was ST11-KPC-2 *K. pneumoniae*. Due to multidrug resistance, and high transmission, ST11-KPC-2 *K. pneumoniae* poses a substantial threat to human health. There were two novel clone groups, ST307 and ST147. ST307 was endemic in Colombia, the United States (Texas), Italy, and South Africa, while ST147 was endemic in India, Greece, Italy, and certain North African countries (Peirano et al., 2020). Due to their ability to cause serious infections, worldwide distribution, and association with AMR, including panresistance, ST307 and ST147 have the ability to become major threats to public health (Peirano et al., 2020), calling for public concern.

Asian countries are the hotbed of NDM producers. This study demonstrated that carrying *bla*_NDM_ genes was the primary mechanism of carbapenem resistance in *Enterobacter spp*. and *E. coli* in southwestern China. Most carbapenemase-producing *E. coli* isolates harbored *bla*_NDM-5_ with high-level carbapenem resistances. All carbapenemase-producing *Enterobacter spp*. strains carried *bla*_NDM-1_ and showed simultaneous resistance to ertapenem, imipenem, and meropenem.

ICU admission and hepatobiliary system disease were identified as independent predictors of CPE infections rather than non-CPE infections. Patients with hepatobiliary system disease might be invaded more frequently by CPE than the non-CPE group. Patients with ICU admission associated with rapidly fatal diseases were at higher risk of acquiring CPE.

In this study, we found that CPE isolates were more resistant to levofloxacin, cefepime, piperacillin/tazobactam, cefoperazone/sulbactam, imipenem, and meropenem than non-CPE isolates. Concerning empiric therapy of patients with CRE infections, there was no significant difference between patients in the CPE group and those in the non-CPE group. About half of the patients with CRE received a carbapenem as a part of the treatment regimen. Generally, clinicians had preferred carbapenem for patients with severe infections. Among definitive therapy of patients with CRE infections, patients with CPE infections received more treament with cefoperazone-sulbactam and/or tigecycline than patients with non-CPE infections. However, CPE isolates were more resistant to cefoperazone-sulbactam than non-CPE isolates. With a tag of CRE infection, patients might receive more advanced but toxic drugs. Therefore, correct identification of CPE or non-CPE is essential for clinical drug selection.

In our study, clinical outcomes were not significantly different between groups with CPE or non-CPE infections. As most CPE isolates were ST11 *K. pneumonia* with *bla*_*KPC-2*_ genes, this comparison is principally between ST11-KPC-2 *K. pneumonia* and a more genetically diverse group of various *Enterobacteriales*. There are some explanations for this finding. First, regardless of the potential carbapenem resistance mechanisms of CRE, CDC standards might identify patients with infections that are associated with poor outcomes. Second, patients attached a label to CRE might receive overtreatment with more toxic drugs that might narrow the difference between the two groups (van Duin et al., 2020). Third, more effective treatment for CPE infections might have weakened the difference in clinical outcomes compared with that of the patients with non-CPE infections. In an observational study, patients with CPE bacteremia demonstrated a more than four times 14-day mortality rate when compared with non-CP-CRE bacteremic patients (aOR 4.92; 95% CI 1.01–24.81) (Tamma et al., 2017). Their study only included cases of CRE bacteraemia. In our study, not only bacteremia but also infections of the respiratory, urinary, catheter, wound, and intra-abdominal sites were included. Patients with CPE infections have a longer ICU stay than the non-CPE group. The reason for the longer ICU stay in the CPE group was unknown. However, this was an important finding suggesting that the identification of a CPE-affected patient indicated outcomes to a greater extent than the identification of a non-CPE patient in our cohort of CRE infection.

There are several limitations in this study. First, due to the small numbers of *K. aerogenes, C. freundii, K. oxytoca, E.kobe, E. coli,* and *E.cloacae*, we only performed an MLST of the most abundant *K. pneumoniae* isolates. Second, our data and isolates were collected from a single centre, where the distribution of carbapenemase genes may differ from the distribution of those in the rest of the world. Third, in any observational study, the analysis of antibiotic treatment regimens was under the influence of confounding biases. Nevertheless, no significant difference of the primary outcome DOOR was observed between the two groups. In China, the most common CPE is ST11-KPC-2 *K. pneumoniae*.

In conclusion, CRE infections were highly associated with poor outcomes, regardless of the CRE subgroup. Patients with CPE infections were associated with prolonged ICU stays and showed different clinical and microbiological characteristics as compared with those with non-CPE infections. Both CPE/non-CPE identification and CRE resistance mechanism determination are essential for better guidance of the clinical administration of patients with CRE infections, especially in those with the history of ICU admission and hepatobiliary system disease.

## Data Availability

The original contributions presented in the study are included in the article/Supplementary Material; further inquiries can be directed to the corresponding authors.
