# Supplementary material for: Clinical and Microbiological Characterization of Carbapenem-Resistant Enterobacteriales: A Prospective Cohort Study
Source: Front Pharmacol. 2021 Oct 8;12:716324. doi: 10.3389/fphar.2021.716324 (PMC8531092; doi:10.3389/fphar.2021.716324)
Supplement: Supplementary file 1 [file DataSheet2.docx]

Table S1. Bacterial species of CRE

|  | ***K*. *pneumonia*** | ***E.spp*** | ***E.coli*** | ***K. aerogene*** | ***K. oxytoca*** | ***C. freundii*** | ***E.kobei*** |
| --- | --- | --- | --- | --- | --- | --- | --- |
| CPE (n=69) | 42(60.9%) | 14(20.3%) | 8(11.6%) | 1(1.4%) | 1(1.4%) | 2(2.9%) | 1(1.4%) |
| Non-CPE (n=59) | 27(45.8%) | 10(16.9%) | 12(20.3%) | 6(10.2 %) | 1(1.7%) | 3(5.1%) | 0(0) |

Data in n (%). CRE=carbapenem-resistant *Enterobacterales*.

CPE= Carbapenemase-producing carbapenem-resistant *Enterobacterales*. Non-CPE=Non-carbapenemase-producing carbapenem-resistant *Enterobacterales*.

Table S2. Isolation sites of CRE

|  | **CPE (n=69)** | **Non-CPE (n=59)** | **Total (n=128)** | **P value*** |
| --- | --- | --- | --- | --- |
| Culture  Blood  Urine  Sputum  Wound  Intra-abdominal Catheter  Drainage  Bile  Other | 11(15.9%)  16(23.2%)  27(39.1%)  4(5.8%)  0(0.0%)  0(0.0%)  6(8.7%)  2(2.9%)  1(1.4%) | 5(8.5%)  18(30.5%)  13(22.0%)  7(11.9%)  2(2.9%)  4(6.8%)  8(13.6%)  3(5.1%)  1(1.7%) | 16(12.5%)  34(26.6%)  40(31.3)  11(8.6%)  2(1.6%)  4(3.1%)  14(10.9)  5(3.9%)  2(1.6%) | **0.042**  0.203  0.350  **0.038**  0.222  0.187  **0.028**  0.379  0.525  0.911 |

Data in n (%). CRE=carbapenem-resistant *Enterobacterales*.

CPE= Carbapenemase-producing carbapenem-resistant *Enterobacterales*. Non-CPE=Non-carbapenemase-producing carbapenem-resistant *Enterobacterales*.

**Table S3.** Susceptibility profiles of CRE

|  | **Isolates susceptible, ^a^n (%)** | | |  |
| --- | --- | --- | --- | --- |
| **Antibotic** | **Total (n=128)** | **CPE (n=69)** | **Non-CPE (n=59)** | **P value** |
| Levofloxacin | 26(20.3%) | 8(11.6%) | 18(30.5%) | **0.008** |
| Imipenem | 29(22.7%) | 2(2.9%) | 27(45.8%) | **<0.001** |
| Meropenem | 33(25.8%) | 3(4.3%) | 30(50.8%) | **<0.001** |
| Ertapenem | 2(1.6%) | 0(0.0%) | 2(3.4%) | 0.211 |
| Ampicillin/sulbactam | 0(0.0%) | 0(0.0%) | 0(0.0%) | >0.999 |
| Piperacillin/tazobactam | 10(7.8%) | 0(0.0%) | 10(16.9%) | **<0.001** |
| Cefmetazole | 0(0.0%) | 0(0.0%) | 0(0.0%) | >0.999 |
| Ceftazidime | 2(1.6%) | 0(0.0%) | 2(3.5%) | 0.211 |
| Cefepime | 7(5.5%) | 0(0.0%) | 7(11.9%) | **0.003** |
| Cefoperazone/Sulbactam | 6(4.7%) | 0(0.0%) | 6(10.2%) | **0.008** |
| Aztreonam | 11(8.6%) | 7(10.1%) | 4(6.8%) | 0.498 |
| Trimethoprim/sulfamethoxazole | 54(42.2%) | 27(39.1%) | 27(45.8%) | 0.449 |
| Tigecycline | 128(100.0%) | 69(100.0%) | 59(100%) | >0.999 |
| Minocycline | 78(60.9%) | 43(62.3%) | 35(59.3%) | 0.729 |

^a^ Including susceptible and intermediate isolates, based on CLSI criteria.

Bold face indicates values that are significant (P<0.05).

Table S4.Carbapenemase genes per species

|  | ***K. pneumonia***  **(n=42)** | ***E.spp* (n=14)** | ***E.coli***  **(n=8)** | ***K. aerogene***  **(n=1)** | ***K. oxytoca***  **(n=1)** | ***C. freundii***  **(n=2)** | ***E.kobe***  **(n=1)** |
| --- | --- | --- | --- | --- | --- | --- | --- |
| KPC-2 | 37(53.6%) | 0 |  | 0 | 0 | 1(1.4%) |  |
| KPC-2;NDM-1 | 0 | 0 |  | 1(1.4%) | 0 |  |  |
| NDM-1 | 0 | 14(20.3%) | 1(1.4%) | 0 | 1(1.4%) | 1(1.4%) | 1(1.4%) |
| NDM-5 | 2(2.9%) | 0 | 6(8.7%) | 0 | 0 |  |  |
| IMP-4 | 3(4.3%) | 0 | 1(1.4%) | 0 | 0 |  |  |

Data in n (%). Only carbapenemase-producing *Enterobacteriaceae* isolates are included.

**Table S5.** Treatments in patients with CRE infections.

|  | **CPE (n=69)** | **Non-CPE (n=59)** | **Total (n=128)** | | **p value** |
| --- | --- | --- | --- | --- | --- |
| **Empiric therapy** |  |  | |  |  |
| carbapennem | 33(47.8%) | 28(47.5%) | | 61(47.7%) | 0.967 |
| carbapennem^a^ | 12(17.4%) | 6(10.2%) | | 18(14.1) | 0.241 |
| piperacillin-tazobactam | 23(33.3%) | 12(20.3%) | | 35(27.3%) | 0.100 |
| cefoperazone-Sulbactam | 23(33.3%) | 16(27.1%) | | 39(30.5%) | 0.446 |
| aminoglycoside | 8(11.6%) | 9(15.3%) | | 17(13.3%) | 0.543 |
| fluoroquinolones | 21(30.4%) | 15(25.4%) | | 36(28.1%) | 0.530 |
| cefoxitin | 18(26.1%) | 9(15.3%) | | 27(21.1%) | 0.134 |
| cefepime | 1(1.4%) | 2(3.4%) | | 3(2.3%) | 0.469 |
| minocycline | 1(1.4%) | 5(8.5%) | | 6(4.7%) | 0.061 |
| tigecycline | 3(4.3%) | 0(0.0%) | | 3(2.3%) | 0.105 |
| glycopeptides | 14(20.3%) | 15(25.4%) | | 29(22.7%) | 0.489 |
| **definitive therapy** |  |  | |  |  |
| carbapennem | 29(42.0%) | 28(47.5%) | | 57(44.5%) | 0.538 |
| piperacillin-tazobactam | 18(26.1%) | 9(15.3%) | | 27(21.1%) | 0.134 |
| cefoperazone-Sulbactam | 20(29.0%) | 7(11.9%) | | 27(21.1%) | **0.018** |
| aminoglycoside | 20(29.0%) | 27(45.8%) | | 47(36.7%) | **0.050** |
| fluoroquinolones | 18(26.1%) | 19(32.2%) | | 37(28.9%) | 0.447 |
| cefepime | 1(1.4%) | 1(1.7%) | | 2(1.6%) | 0.911 |
| minocycline | 11(15.9%) | 6(10.2%) | | 17(13.3%) | 0.337 |
| tigecycline | 25(36.2%) | 11(18.6%) | | 36(28.1%) | **0.027** |
| glycopeptides | 11(15.9%) | 13(22.0%) | | 24 (18.8%) | 0.379 |

Data are expressed as n (%) of patients for categorical variables.

Bold face indicates values that are significant (P<0.05).

^a^ Monotherapy with a carbapenem
