# Supplementary material for: Clinical and Microbiological Characterization of Carbapenem-Resistant Enterobacteriales: A Prospective Cohort Study
Source: Front Pharmacol. 2021 Oct 8;12:716324. doi: 10.3389/fphar.2021.716324 (PMC8531092; doi:10.3389/fphar.2021.716324)
Supplement: Supplementary file 2 [file DataSheet1.DOCX]

Supplementary Materials

**Clinical Definitions**

Critical illness was determined as a Pitt bacteremia score ≥4 points on the day of culture [1]. The Charlson comorbidity index (CCI) was calculated based on chart abstraction [2]. Infections were defined by previously described standard criteria [3].

**Outcome Definitions**

If the discharge date was prior to 30 days after index culture date, symptomatic response was defined as either resolution or improvement of symptoms at the time of discharge. If the discharge date was later than 30 days after the index culture date, culture-type specific criteria were used to determine response. Clinical response was defined as a symptomatic response without need for ongoing treatment with any antibiotics with in vitro activity against the index CDC-defined CRE and without relapse. Relapse was defined as a new culture episode from the same anatomical site with the same species of CRE within 30 days of the date of the index first positive culture. “Unsuccessful discharge” was defined as either a post-culture hospital stay of ≥30 days or documented readmission within 30 days. Post-culture renal failure was defined by RIFLE criteria as any three-fold or greater increase in serum creatinine from the level on the day of index culture until discharge [4]. In addition, patients who newly required renal replacement therapy after the index culture date were deemed to have post-culture renal failure. Patients who were on renal replacement therapy prior to hospitalization and/or during hospitalization but prior to the index culture were not eligible to develop postculture renal failure. Patients were deemed to have an adverse event if they had a *Clostridioides difficile* infection and/or post-culture renal failure.

**Supplementary references**1. Chow JW, Yu VL. Combination antibiotic therapy versus monotherapy for gram-negative bacteraemia: a commentary. *Int J Antimicrob Agents* 1999; **11**(1): 7-12.
2. Charlson ME, Pompei P, Ales KL, et al. A new method of classifying prognostic comorbidity in longitudinal studies: development and validation. *J Chronic Dis* 1987; **40**(5): 373-83.
3. van Duin D, Perez F, Rudin SD, et al. Surveillance of Carbapenem-Resistant *Klebsiella pneumoniae*: Tracking Molecular Epidemiology and Outcomes through a Regional Network. *Antimicrob Agents Chemother* 2014; **58**(7): 4035-41.
4. Bellomo R, Ronco C, Kellum JA, et al. Acute Dialysis Quality Initiative workgroup. Acute renal failure - definition, outcome measures, animal models, fluid therapy and information technology needs: the Second International Consensus Conference of the Acute Dialysis Quality Initiative (ADQI) Group. Crit Care. 2004 Aug; 8(4):R204-12.
